# Helminth and Host Crosstalk: New Insight Into Treatment of Obesity and Its Associated Metabolic Syndromes

**DOI:** 10.3389/fimmu.2022.827486

**Published:** 2022-02-25

**Authors:** Mengyu Dai, Xiaoying Yang, Yinghua Yu, Wei Pan

**Affiliations:** ^1^ Jiangsu Key Laboratory of Immunity and Metabolism, Department of Pathogen Biology and Immunology, Xuzhou Medical University, Xuzhou, China; ^2^ The Second Clinical Medicine, Xuzhou Medical University, Xuzhou, China; ^3^ National Demonstration Center for Experimental Basic Medical Science Education (Xuzhou Medical University), Xuzhou, China

**Keywords:** parasite, obesity, metabolic inflammation, macrophages, fat browning, adipokine, insulin resistance, microbiota

## Abstract

Obesity and its associated Metabolic Syndromes (Mets) represent a global epidemic health problem. Metabolic inflammation, lipid accumulation and insulin resistance contribute to the progression of these diseases, thereby becoming targets for drug development. Epidemiological data have showed that the rate of helminth infection negatively correlates with the incidence of obesity and Mets. Correspondingly, numerous animal experiments and a few of clinic trials in human demonstrate that helminth infection or its derived molecules can mitigate obesity and Mets *via* induction of macrophage M2 polarization, inhibition of adipogenesis, promotion of fat browning, and improvement of glucose tolerance, insulin resistance and metabolic inflammation. Interestingly, sporadic studies also uncover that several helminth infections can reshape gut microbiota of hosts, which is intimately implicated in the pathogenesis of obesity and Mets. Overall, these findings indicate that the crosstalk between helminth and hosts may be a novel direction for obesity and Mets therapy. The present article reviews the molecular mechanism of how helminth masters immunity and metabolism in obesity.

## Introduction

Obesity, an epidemic and systemic metabolic disease, is characterized by excessive fat accumulation and low-grade chronic inflammation. The prevalence of obesity is increasing at an alarming rate in many parts of the world since 1975, arising public awareness (1). Notably, obesity is well recognized to increase the risk of Metabolic Syndromes (Mets) including type 2 diabetes (T2DM), cardiovascular diseases (CVD), non-alcoholic fatty liver diseases (NAFLD) and other metabolic disorders (2, 3). Particularly, accumulating evidence links obesity as a crucial factor with neurodegenerative diseases such as Alzheimer’s disease (AD) (4), which still lacks effective therapy, although numerous money has been invested in this area (5). Considering the grave consequences, it is therefore imperative to seek novel strategies against obesity and its associated Mets.

Inappropriate diets have been identified as the key factor for obesity and Mets. The findings from animal experiments and population epidemiological investigations firmly support that the long-term intake of western diet (WD) is closely associated with the incidence of obesity and Mets (6–8). As an extremely active endocrine organ in the body, adipose tissue can release a large variety of adipokines that regulate diverse biological processes such as glucolipid metabolism, energy expenditure, appetite control, insulin sensitivity, and inflammation (9). In obese state, excessive fat accumulation produces multiple metabolites including palmitic acid (PA) and adipokines, which activate pro-inflammatory pathways and release inflammatory factors (10, 11). They can jointly promote the progression of obesity and Mets. Interestingly, recent studies have uncovered that gut microbiota is also implicated in the pathogenesis of obesity, diabetes and neurological disorders (12, 13). In view of the essential role of these factors mentioned above in obesity and Mets, reprogramming these key events therefore benefits for development of intervention strategies.

Parasites are categorized into protozoa (*Trypanosoma*, *Toxoplasma*, *etc.*) and helminths (cestodes, nematodes, trematodes). As one of the relatively successful pathogens, helminth infects approximately a quarter of the world’s population, seriously endangering public health and causing social and economic problems. However, the mortality due to helminth infection is rare, suggesting a long evolutionary co-adaptation between parasites and human. Helminth is recognized to be the strongest natural stimuli of type-2 immune responses, which can down-regulate the anti-infective immunity, thereby allowing the long-term survival of the parasites in hosts (14–16). In recent years, epidemiological evidence shows that there is an inverse correlation between the exposure to helminth and the prevalence of obesity and Mets (17–21). The Hygiene Hypothesis proposes that the fewer infections (especially helminth infection) in early childhood lead to the greater possibilities of developing allergic, inflammatory and metabolic diseases in the future, which implies the ability of helminths to master immunopathology and the potential therapeutic effects on diseases (22, 23). According to published studies, the infections of several helminths have been reported to alleviate obesity and Mets *via* inhibition of adipogenesis, improvement of glucose tolerance and insulin resistance (IR) (24–27). Furthermore, accumulating studies have showed that helminth derived molecules or excretory-secretory products (ESPs) can act as key modulators to exert metabolic and immune modifying functions (28). For example, ES-62, a protein secreted by filarial nematode *Acanthocheilonema viteae*, is reported to prevent metabolic dysfunction *via* promotion of the recruitment of eosinophils and M2 type macrophages in retroperitoneal adipose tissues of infected mice (29–31). Notably, not all parasitic infection is beneficial for improving metabolic disorders. Infection stage and parasite species are the key factors that determine the protective or harmful effect in the condition of obesity. It is recently reported that *Trypanosoma cruzi* (*T. cruzi*) infection induces adipogenic signaling and promotes the accumulation of lipid in the hearts during the early chronic stage in infected mice (32). Moreover, the parasite can exacerbate inflammation and aggravate obesity related metabolic disorders (*e.g.* atherosclerosis and NAFLD) during the acute phase of infection in obese mice, due to a high affinity for host cholesterol (33, 34). Since the relationship between protozoa and adipose tissue has been reviewed (25, 35), we herein did not have too much discussion about it. In the present article, we mainly focused on the underlying mechanism of how helminth infection or their derived molecules reprogram metabolic inflammation, fat browning, adipokine production, IR and gut microbiota in WD-induced obesity, which could provide a basis for helminth therapy against obesity and Mets.

## Helminth-Induced M2 Macrophage Polarization *via* Metabolic Reprogramming Creates an Anti-Inflammatory Environment

Macrophages, one of dominant immune cells in adipose tissues, have the characteristics of pluripotency and plasticity that can differentiate into different phenotypes after exposure to endogenous or exogenous stimuli. In obese mice, PA, lipopolysaccharide (LPS), and tumor necrosis factor-α (TNF-α) can polarize macrophages towards “classically activated” phenotype (M1 type) that releases pro-inflammatory cytokines (such as IL-6, TNF-α, IL-1β) (10, 36, 37). In lean adipose tissues, resident macrophages are alternatively activated, exhibiting an anti-inflammatory and M2-like phenotype that involves in homeostasis maintenance of adipose tissues (38). Macrophage polarization is featured by the change of cell surface marker expression. CD80 and CD86 are universally acknowledged markers for M1 macrophages. In contrast, the level of arginase-1 (Arg-1), mannose receptor (CD206), and chemokine (C-C) motif ligand 17 (CCL17) and CCL22 are significantly increased in M2 macrophages (39). M1 macrophages sense intracellular pathogens mainly through the expression of toll-like receptors (TLRs) (40), whereas M2 macrophages sense extracellular pathogens through expression of scavenger receptors (41). In infected tissues, pro-inflammatory M1 phenotype are first polarized to protect the host against pathogens, followed by M2 polarization to form an anti-inflammatory response and promote tissue repair. M2 macrophages are reported to participate in eliminating dead adipocytes for the melioration of inflammatory milieu, and recruiting adipocyte progenitors for the regulation of their proliferation and differentiation, finally controlling fat hypertrophy as well as effectively improving obesity (42). Therefore, targeting adipose tissue inflammation and inducing M2 macrophages have emerged as potential therapeutic strategies for obesity-related metabolic disorders (43).

The helminth infection is accompanied by the setting of complex metabolic reprogramming events and the induction of macrophages M2 polarization in adipose tissues (44, 45), which provides a basis for obesity intervention. Prior observations have reported that *Heligmosomoides polygyrus* (*H. polygyrus*) infection induces polarization of M2 macrophages with upregulation of anti-inflammatory cytokines (Arg1, IL-10), to resist metabolic inflammation mediated by M1 macrophages in WD fed mice (46, 47). Moreover, WD mice had less body weight gain after administration of *H. polygyrus*-induced M2 macrophages (46). Similarly, Cortes-Selva et al. found that *Schistosoma mansoni* (*S. mansoni*) infection induces M2 polarization of macrophages to improve hyperlipidemia and atherosclerosis (48). Interestingly, acute *T. cruzi* infection potentiates adipose tissue inflammation accompanied by M1 macrophage infiltration (49), whereas chronic *T. cruzi* infection can cause a shift in the M2/M1 ratio towards an anti-inflammatory phenotype (35, 50). Besides live parasite infection, their ESPs or derived molecules can regulate macrophage polarization. Omega-1, one of major immunomodulatory glycoproteins in the eggs of *S. mansoni*, induces type 2 immune response, and improves metabolic homeostasis through independent inhibition of food intake in WD fed mice (51). Moreover, Hussaarts et al. reported that chronic *S. mansoni* infection and its soluble egg antigens (SEA) promote the infiltration of eosinophils and the accumulation of M2 macrophages in adipose tissues, thereby ameliorating WD-induced obesity (24).

As the component of ESPs released by helminth, extracellular vesicles (EVs), a group of heterogeneous lipid-enclosed particles derived from different cells ranging from nano to micrometer in size, have emerged as a new mediator for intercellular communication (52). Notably, EVs derived from helminths have spurred a new paradigm in studying host-helminth interaction (53). Several studies have showed that parasite derived EVs can regulate pro- or anti-inflammatory responses and induce macrophage polarization (54). For example, *Echinococcus multilocularis* EVs is reported to trigger production of anti-inflammatory cytokines by activation of M2 macrophages (55). *Trichinella spiralis* EVs can improve colitis *via* induction of M2 macrophages infiltration (56). Importantly, a growing body of findings highlight that adipose tissue-secreted EVs can maintain metabolic homeostasis through polarizing M2 macrophages, inhibiting adipocyte hypertrophy and promoting fat browning, which ameliorates obesity and Mets (57, 58). Thus, it is most likely that helminth EVs may be a novel direction to discover intervention strategies for obesity and Mets.

**Figure 1:**
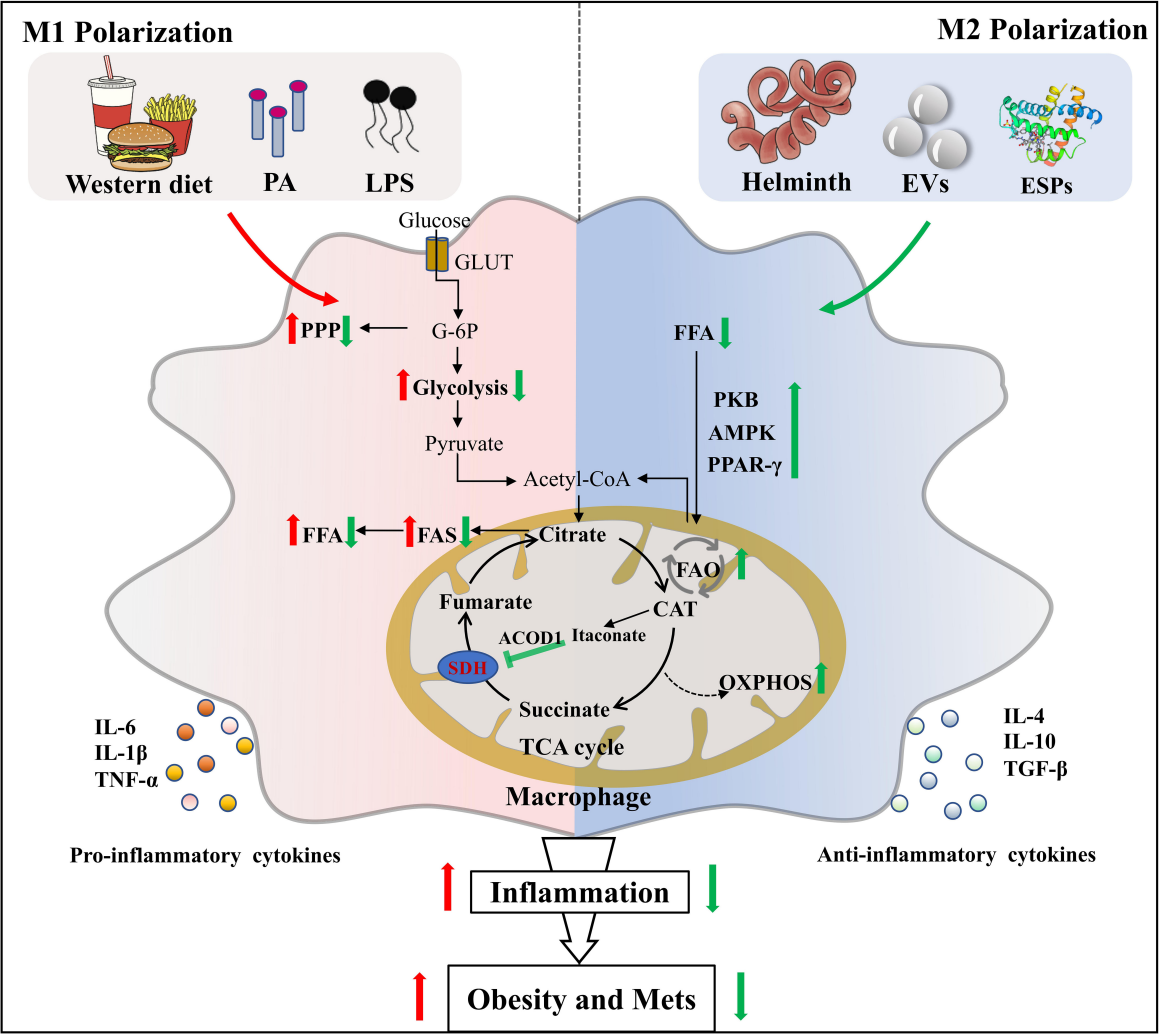
Helminth or its derived molecules induce M2 polarization of macrophages *via* metabolic reprogramming in obesity. Helminth infection or their derived molecules can induce M2 macrophages polarization along with the setting of complex metabolic reprogramming events in adipose tissue of mice fed by western diet, which provides therapeutic potential for obesity and Mets. PA, palmitic acid; LPS, lipopolysaccharide; GLUT, glucose transporter; G-6P, glucose-6-phosphate; PPP, pentose phosphate pathway; FFA, free fatty acid; FAS, fatty acid synthesis; SDH, succinate dehydrogenase; ACOD1, aconitate decarboxylase 1; CAT, cis-aconitate; EVs, extracellular vesicles; ESPs, excretory-secretory products; PKB, protein kinase B; AMPK, adenosine monophosphate (AMP)-activated protein kinase; PPAR-γ, peroxisome proliferator–activated receptor-γ; FAO, fatty acid oxidation; OXPHOS, oxidative phosphorylation.

In recent years, the emerging immunometabolism has attracted considerable research interest (59, 60). The discipline shows that the changes in intracellular metabolic pathways can determine the differentiation and effector function of immune cells including macrophages (61). M1 macrophages express high levels of glycolysis and pentose phosphate pathways, whereas M2 macrophages depend on mitochondrial respiration and oxidative phosphorylation (OXPHOS) for energy supply (62). During the chronic or late stage of helminth infection, the host immune response is characterized by type 2 immune response, in which M2 macrophage is dominant. Helminth infection differentiates macrophages into M2 phenotype through increasing expression of IL-4 and maintaining the IL-4 signaling pathways (*e.g.* fatty acid oxidation, FAO and OXPHOS) (63–65). It is well established that LPS-induced M1 activated cells are characterized by elevated glycolysis rate, enhanced pentose phosphate pathway, and attenuated OXPHOS level (66). Interestingly, *Trypanosoma. brucei* metabolite indolepyruvate can inhibit this effect mentioned above, and decrease the pro-inflammatory cytokine IL-1β production in macrophages, thereby contributing to immune evasion (67). Concurrently, we previously found that mice infected with the larval *Echinococcus granulosus* (*E. granulosus*) show enhanced lipolysis in adipose tissue, which is accompanied by increased arginine metabolism (26). It is generally known that arginine metabolism has an intimate association with M2 polarization. A recent study also discovers that blockage of tricarboxylic acid cycle (TCA cycle) can reprogram metabolic flux, resulting in the accumulation of metabolites such as succinate and fumarate, which in turn act as metabolic signals to modulate macrophage function (68). Succinate has been demonstrated to hyperpolarize M2 macrophages *via* interacting with its receptor succinate receptor 1 (SUCNR1) (69–71). It has been reported that succinate level is elevated prior to host cells invasion by *T. cruzi* (72), but the association between succinate in *T. cruzi* infection and M2 macrophages polarization remains to be elucidated. Succinate dehydrogenase (SDH) is recently demonstrated to be a major energetic metabolic node and a crucial regulator of activation of M1 macrophages. SDH inhibition is found to cut off pro-inflammatory signal in mitochondria, thereby driving anti-inflammatory phenotype (73). For instance, Lampropoulou et al. found that itaconate, one of the most easily induced metabolites in activated macrophages, exerts anti-inflammatory effects by suppressing SDH-catalyzed oxidation of succinate (74, 75). Of note, SDH enzyme activities were significantly decreased after *S. mansoni* infection (76), causing succinate accumulation followed by preventing the induction of a range of pro-inflammatory factors (IL-1β) and enhancing a range of anti-inflammatory factors (IL-1RA and IL-10) (73). Thus, helminth infection may promote M2 polarization of macrophages by reprogramming metabolism (**Figure 1**). Exploitation of M2 macrophages induced by parasitic infection to inhibit metabolic inflammation and adipogenesis may provide a niche for the intervention of obesity and Mets.

## Helminth Infection May Recruit Other Immune Cells to Regulate Homeostasis of Metabolism and Immunity

**Figure 2:**
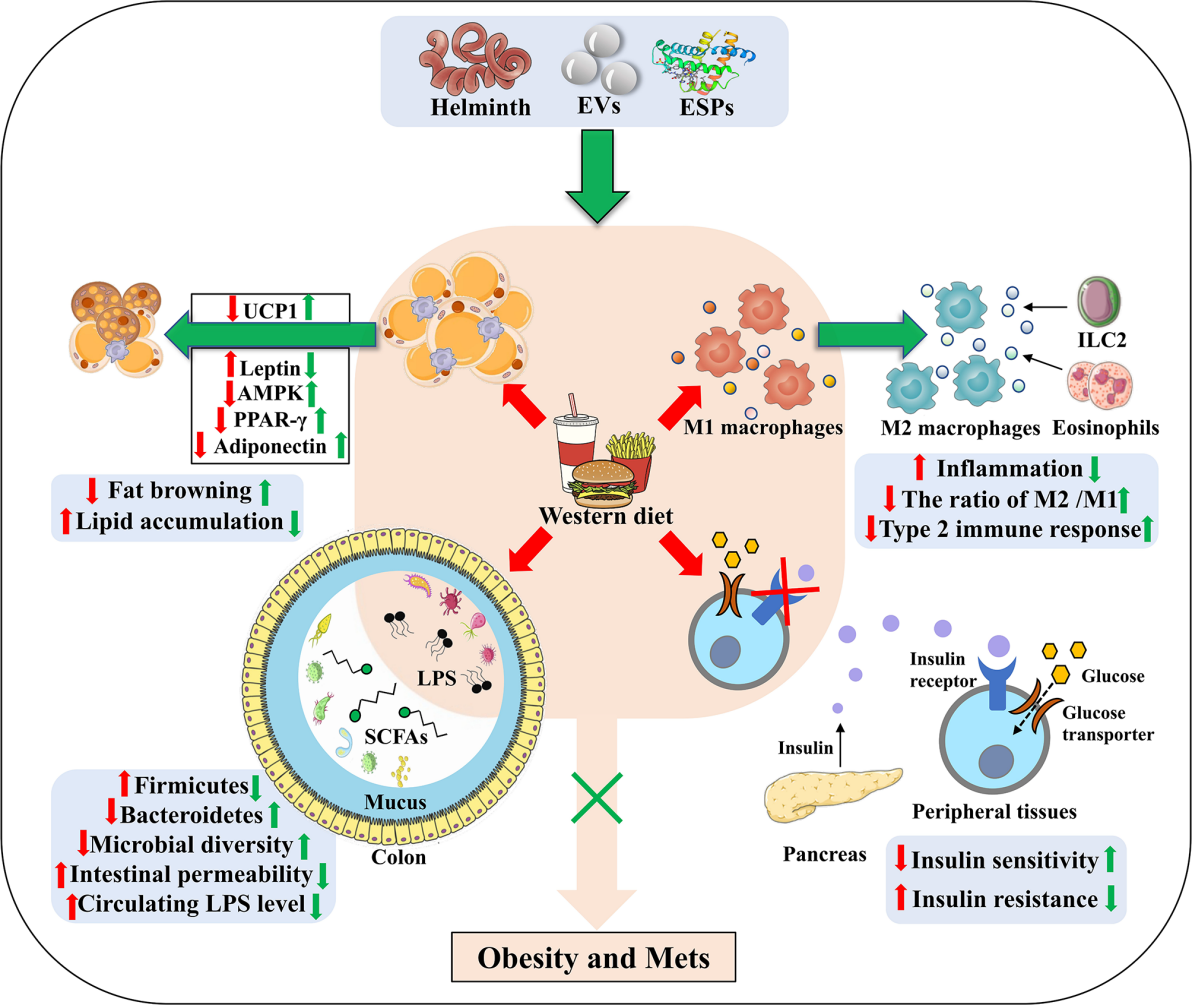
The underlying strategies of how helminth or its derived molecules modulate the tissue-specific homeostasis in obesity. Helminth infection or their derived molecules can ameliorate western diet-induced obesity and its associated Mets through inducing M2 macrophage polarization, down-regulating metabolic inflammation, promoting fat browning, attenuating lipid accumulation, ameliorating insulin resistance (through improving the impaired ability of glucose uptake and promoting insulin binding to its receptors) and relieving the dysbiosis of gut microbiota. EVs, extracellular vesicles; ESPs, excretory-secretory products; UCP1, uncoupling protein 1; AMPK, adenosine monophosphate (AMP)-activated protein kinase; PPAR-γ, peroxisome proliferator–activated receptor-γ; LPS, lipopolysaccharide; SCFAs, short-chain fatty acids; ILC2, group 2 innate lymphoid cells.

In adipose tissues, T cells, B cells and eosinophils are also important mediators for homeostasis of metabolism and immunity. Generally, T helper1 (Th1) cells, Th17 cells, CD8^+^ T cells and B2 cells are responsible for the obesity-induced inflammation, while regulatory T cells (Tregs), regulatory B cells (Bregs), Th2 cells, B1 cells, and eosinophils contribute to the anti-inflammatory response (77, 78). However, the balance is often broken after long term intake of WD, which is accompanied by IR (79, 80). Several studies have showed that helminth infection or ESPs can suppress the differentiation of Th1 and Th17, and promote the induction of Tregs and Bregs to resist the anti-infectious immunity (16, 81, 82). It is reported that S*.mansoni* infection alleviates allergy airway inflammation *via* induction of Treg population (83, 84). Moreover, induction of Bregs post *Schistosoma japonicum* (*S. japonicum*) regulates the systematic inflammation induced by WD (85). In addition, *S. japonicum*-activated Bregs can control the levels of proinflammatory chemokines and cytokines by IL-10 secretion to protect against the parasite induced liver inflammation and fibrosis (86). Finally, chronic *S. mansoni* infection and SEA injection promote eosinophil activation *via* IL-4 secretion to maintain M2 macrophages polarization in adipose tissue (24) (**Figure 2**). Thus, it is rational that these anti-inflammatory cell populations also contribute to the beneficial effect of helminth infection on obesity and Mets, although the underlying mechanism has not been investigated.

## Helminth Infection Promotes Fat Browning *via* Elevation of UCP1 Expression

Adipose tissue, which can maintain a dynamic balance between energy storage in the form of lipids and energy utilization (87), has been traditionally subclassified into white adipose tissue (WAT) and brown adipose tissue (BAT) based on morphological and functional difference. WAT, the main site for the body to store lipids, stores excess energy in the form of triglycerides; whereas, BAT plays an important role in regulating energy balance and protecting against obesity by virtue of the capability for energy expenditure through thermogenesis mediated by a BAT-specific mitochondrial protein, uncoupling protein1 (UCP1) (88, 89). Recent data suggest that there are two distinct types of BAT: classical BAT derived from a myogenic factor 5 (myf-5) cellular lineage, and “brown-like” cells that reside in WAT from a non-myf-5 lineage, also called beige or brite cells (90). When mice are chronically exposed to cold environment, β3-adrenergic receptor agonists or peroxisome proliferator–activated receptor (PPAR)-γ agonists (90), the pre-existing beige adipocytes will go through phenotypic “transdifferentiation”, and “fat browning” (a switch from energy-storing white adipocytes to thermogenic brown fat-like cells) will occur (91–93). In contrast to extremely low basal expression of UCP1 within WAT, the UCP1 expression in beige adipocytes after exposure to the same stimuli is upregulated to the levels that can resemble classic brown adipocytes, which is consistent with increased BAT and a greater capacity for energy dissipation through thermogenesis. The potential contribution of BAT thermogenesis and fat browning to whole body energy expenditure, consequently, can be considered as a therapeutic target to combat obesity and its related comorbidities (94, 95).

It is reported that *H. polygyrus* infection elevates UCP1 expression, promoting the browning of WAT in mice, by which can increase energy expenditure and attenuate obesity in mice (46). Moreover, *S. mansoni* egg-derived ω1, a predominant type 2-inducing molecule, induces systemic and localized release of the type 2 initiator cytokine IL-33 that is involved in maintaining glucose homeostasis and promoting browning of WAT (96). In addition, group 2 innate lymphoid cells (ILC2s) activated by IL-33 are demonstrated to produce methionine-enkephalin peptides and catecholamines, which can directly upregulate UCP1 expression in adipocytes, thereby enhancing fat browning and improving metabolic parameters in obese mice (97–99). Helminth infection, therefore, holds the promise for increasing fat browning to ameliorate obesity (**Figure 2**).

## Helminth Infection Alters the Levels of Adipokines and the Expression of Enzymes in Lipid Metabolism

Far from hormonally inert, adipose tissue has been, in recent years, recognized as a major endocrine organ, as it produces a wide spectrum of adipokines such as adiponectin, leptin and resistin that play a role in glucolipid homeostasis and immune regulation (100–103). Notably, leptin and resistin have pro-inflammatory effects *via* increasing the production of IL-6 and other pro-inflammatory factors, whilst adiponectin exerts anti-inflammatory properties due to the inhibition of TNF-α (104). Leptin, a hormone that is capable of effectively reducing food intake and body weight, was initially considered for obesity treatment. Indeed, obese mice have since been found to exhibit higher leptin mRNA levels, directly associated with the increased adiposity (101). Due to defects in the blood-brain barrier transduction pathway, obese mice often develop hyperleptinemia and central leptin resistance (105, 106). The inability of leptin to exert its anorexigenic effects in obese individuals, and therefore, the lack of clinical utility of leptin in obesity, is defined as leptin resistance (107). It has been reported that chronic *T.cruzi* infection can improve leptin resistance in obese mice (50, 106). Additionally, leptin and other adipocytokines can jointly induce the recruitment and activation of immune cells during WAT expansion in obese mice, create a pro-inflammatory environment and promote the release of free fatty acids, so as to exacerbate obesity-associated metabolic inflammation (50) (**Figure 2**). When obese mice were infected with *H. polygyrus*, the gene expression of leptin was markedly decreased (46), thereby reducing the production of fatty acids and fighting against obesity.

The physiological function of adipose tissue depends on fat synthesis and lipolysis, and both the progresses are strictly manipulated by local adipokines (such as PPAR-γ, adiponectin, TNF-α) (108, 109). When mice were infected with *T. cruzi* at an acute phase, adipose tissue displayed a significant decrease of lipid accumulation, adipocyte size and fat mass, which was correlated with increased expression of lipolytic enzymes (49). As a dominant transcription factor of adipogenesis and a master modulator of adipocyte differentiation, PPAR-γ promotes lipid storage in adipose tissue through stimulating the expression of lipogenic enzymes and inhibits the secretion of inflammatory mediators when activated by the PPAR-γ agonist (110). González FB et al. showed that experimental acute *T. cruzi* infection downregulated PPAR-γ expression (50, 111), coming to a state compatible with the adipose tissue atrophy and M1 macrophage polarization (112). Moreover, *H. polygyrus* infection may ameliorate diet-induced obesity *via* modulating gene expression of key transcription factors in adipogenesis, such as PPAR-γ and CCAAT enhancer-binding proteins α (C/EBPα) (46). Nevertheless, *Schistosomal*-derived lysophosphatidylcholine can induce M2 macrophage polarization secondary to increased PPAR-γ expression (113). It is reported that adenosine monophosphate (AMP)- activated protein kinase (AMPK), a potent cellular energy sensor for maintenance of metabolism homeostasis, can favor FAO and limit fatty acid synthesis to regulate lipid accumulation (114). Xu et al. reported that *S. japonicum* infection can exert a strong metabolic effect *via* activating the AMPK and protein kinase B (PKB, also known as AKT) signaling molecules in *S. japonicum* SEA-stimulated macrophages, which further promote FAO and suppress fatty acid synthesis (115). Moreover, *S. japonicum* infection induces upregulated expression of the FAO-related genes while downregulating the expression of the genes associated with fatty acid synthesis and lipid uptake, which is consistent with SEA-induced anti-inflammatory M2 phenotype (116, 117). It is reported that M2 macrophages shifts into FAO and OXPHOS states, directed by signaling *via* IL-4 (118). Therefore, parasite infection or its derived molecules may reduce fat mass and improve obesity by regulating lipid metabolism in infected hosts (**Figure 2**).

## Helminth Infection Ameliorates Insulin Sensitivity and Insulin Resistance

In obesity, abnormal adipokine secretion and excessive lipid accumulation can cause a decrease in the expression or activity of glucose transporter 4 (GLUT4) *via* phosphoinositide 3-kinase (PI3K)/AKT signaling pathway. Alterations in GLUT4 translocation impair glucose uptake and insulin sensitivity, finally contributing to IR (119–122) (**Figure 2**). IR is characterized by glucose dysregulation with elevated serum insulin level, which increases the risk for metabolic syndromes such as T2DM, CVD and polycystic ovary syndrome (PCOS) (122–125). Recent data have showed that in obese animals, proinflammatory mediators (namely TNF-α, IL-6, and IL-1β) can damage the pancreatic β cells insulin secretion function in autocrine and paracrine manners, and down-regulate insulin sensitivity in liver and skeletal muscle, jointly inducing the occurrence of IR that is closely related to the development of T2DM (126–128).

Emerging evidence has demonstrated that parasitic infection can improve IR and glucose tolerance in obese mice (129). Eosinophils are reported to improve glucose homeostasis by inducing macrophage M2 polarization in adipose tissue of obese mice (130). In line with this, *Nippostrongylus brasiliensis* (*N. brasiliensis*) infection can increase percentages of ILC2s and accumulation of eosinophils in visceral adipose tissues to enhance insulin sensitivity (131). Simultaneously, infection of obese mice with *N. brasiliensis* can attenuate body weight gain, decrease adipose tissue mass, and ameliorate glucose metabolism and insulin sensitivity, accompanied by a dramatic decline of insulin levels (132). In addition, Filarial nematode *Litomosoides sigmodontis* (*L. sigmodontis*) infection and *L.sigmodontis* antigen (LsAg) administration is reported to increased numbers of eosinophils and M2 macrophages within adipose tissues and improve glucose tolerance in obese mice by the eosinophil-dependent mechanism (133). Notably, LsAg administration can also increase the level of adiponectin that related to insulin sensitization and inhibit the expression of proinflammatory factor interferon-γ (IFN-γ) and IL-17 to improve IR (134). Obese mice treated with LsAg injections show significant upregulation of gene expression that are linked to insulin sensitivity, such as GLUT4 and hexokinase 2 (HK2), which may further support insulin signaling and improve IR (133). Furthermore, administration of lacto-N-fucopentaose III (LNFPIII), an immunomodulatory glycan derived from *S. mansoni* SEA, is shown to improve insulin sensitivity and glucose tolerance in obese mice, which is mediated partly *via* IL-10 production in macrophages and dendritic cells (51, 135). It is well recognized that high level of IL-10 reduces the risk for Mets, particularly T2DM (136). Correspondingly, a cross-sectional study performed by Hays et al. showed a negative correlation between the infection rate of *Strongyloides stercoralis* and the occurrence of T2DM (137). As a consequence, helminth infection can improve IR and prevent obesity-related Mets (**Figure 2**).

## Helminth Infection Reshapes the Composition of Gut Microbiota

The gut microbiota act as an important factor in the progression of obesity *via* maintenance of energy homeostasis and host immunity (138, 139). It has been reported that the decrease in the richness and diversity of gut microbiota in obese mice induced by WD is accompanied by a reduction in expression of intestinal tight junction proteins, which is linked to increased intestinal permeability, thereby resulting in a malfunctioning gut barrier (140) (**Figure 2**). Moreover, increased circulating LPS levels due to a “leaky gut”, can induce an inflammatory state and metabolic hyperendotoxinemia, eventually driving the development of obesity-associated IR and cognitive impairment (141–143). The association between the composition of the gut microbiota and metabolic dysfunction is becoming clear and has been extensively reported. Therefore, modulation of the gut microbiota may be a potential therapeutic way for treating obesity and Mets (144).

*H. polygyrus* infection can induce significant alterations in gut microbiome composition as evidenced by a marked increase in *Bacteroidetes* and a decrease in *Firmicutes* (145). Similarly, Walk ST et al. found a significant shift in the abundance and relative distribution of bacterial species in the ileum of mice post *H. polygyrus* infection (146). *H. polygyrus*-modulated microbiota exhibit levels of short-chain fatty acids (SCFAs) and upregulate expression of G protein coupled receptors (GPRs) (145). SCFAs (mainly acetate, butyrate and propionate), the key bacterial metabolites, can participate in host energy homeostasis and immune function, playing a beneficial role in preventing obesity *via* interacting with GPRs. It is reported that acetate can improve appetite control through the interaction with the central nervous system (147), and that butyrate and propionate can not only induce the production of gut hormones associated with the reduction of food intake, but also enhance gut epithelial barrier integrity as well as promote an anti-inflammatory milieu (148–150). A recent study also shows that SCFAs-induced protection against HFD-induced obesity is mediated by down-regulation of PPARγ, promoting a switch from lipid synthesis to lipid oxidation (151). Moreover, *H. polygyrus* affects the composition of the intestinal microbiota to increase norepinephrine and then enhance UCP1 expression in adipose tissues, which is responsible for limiting weight gain (152). Infection with *Strongyloides venezuelensis* results in modifications of the gut microbiota, most notably by increasing *Lactobacillus* spp. These modifications in the microbiota may alter host metabolism by switching macrophages from M1 to M2 in the adipose tissue, increasing the levels of anti-inflammatory cytokines, upregulating the expression of tight junction proteins (thereby reducing the permeability) and decreasing LPS in the sera. Furthermore, these changes correlate with improved insulin signaling and sensitivity, suggesting that modulation of the microbiota by helminth infection has a positive effect on the glucose homeostasis of hosts (153). In line with others reports, our latest work showed that the ESPs derived from the larval *E. granulosus* improves cognitive decline, mitigates the gut microbiota dysbiosis, and reverses gut barrier dysfunction in WD fed mice (154). Notably, ablation of gut microbiota abolishes the effect of ESPs on brain and gut. This is the first time to utilize parasite model to treat obesity induced cognitive decline *via* microbiota-gut-brain axis (154). It is therefore proposed that helminth or its derived molecules-induced alterations of microbiota composition, and microbiota-produced metabolites may play a vital but neglected role in the protective effects of helminth infection on obesity (**Figure 2**).

## Summary and Prospect

As one of relatively successful pathogens, parasites have coevolved with human over millennia, developing elegant and intricate immune escape mechanisms to manipulate the equilibrium of immune and metabolism in hosts. Helminth infection or their derived molecules (mainly ESPs) can lead to a broad range of outcomes that ameliorate WD-induced obesity and metabolic disorders, including M2 polarization of macrophages (**Figure 1**), down-regulation of metabolic inflammation by triggering the release of type 2 cytokines (such as IL-4, IL-10, and IL-13), the increase of thermogenesis and energy consumption by promoting fat browning, modification of adipokines and lipid metabolism, improvement of insulin sensitivity and glucose tolerance, and modulation of gut microbiota (**Figure 2**).

Fully understanding and harnessing characteristics of helminth-driven immunomodulation may become an important therapeutic insight for human diseases including obesity and Mets (155). In 2013, the safety and tolerability of *Trichuris suis* ova (TSO) have been confirmed in randomised clinical trials (156), and the United States Food and Drug Administration has approved TSO to treat patients with inflammatory bowel diseases (IBD). A recent randomized, double-blind, placebo-controlled Phase 1b clinical trial conducted by Pierce et al. (157), showed that it is safe and well-tolerated to inject a certain dose of larvae III stage of *Necator americanus* into patients with central obesity and metabolic syndrome, identifying hookworm infection as a potentially alternative therapy for obesity. Overall, these clinical trials lay a foundation for further exploiting helminth-inspired therapies against obesity and Mets.

However, helminth therapy also presents a challenge to the drug development community. This results from the fact that live parasitic administration has the possibility to increase the infective risk and bring biosafety issues. Alternatively, animal models have demonstrated that the ESPs released by parasites can evoke type 2 immune responses, alleviate adipose tissue inflammation and enhance glucose homeostasis, thereby reducing the body weight of obese animals (155). The use of ESPs instead of active helminth infection potentially addresses some of the drawbacks and obstacles currently faced by experimental helminth therapy. However, type 2 immunity induced by parasite derived molecules may also have adverse effects. For example, a higher occurrence rate of asthma is observed in obese individuals (158, 159), while the tropomyosin of *Ascaris lumbricoides* is reported to have strong allergenic activity (160). Administration of such molecules is speculated to increase the incidence rate of asthma in obesity. What’s more, it is well known that parasite infection is closely associated with accumulation of eosinophils (24, 131), and eosinophil recruitment represents one of the pathogenesis for asthma (161). Thus, to frame the possibility that helminth derived molecules could be developed as drugs, more animals and clinical trials should be tested.

Furthermore, a more elaborated description of the definitive immunomodulatory components of helminth could facilitate a more precise therapeutic approach against obesity and its associated Mets. The molecular diversity of helminth products with therapeutic potential is noticeable. As such, identifying specific molecules, targeted receptors, and downstream signaling pathways that work in therapy or prevention of obesity and its related Mets, constitutes important future directions. Even if the molecules involved in their immunometabolic effects are identified, immune suppression to human should be avoided, in order to prevent other complications. Of note, recombinant expression platform is overwhelmingly vital for the production of developing helminth biologics. Moreover, if helminthic therapy comes true as innovative therapeutic avenues for obesity and its associated disorders, the drug dose, the frequency and route of administration and duration of treatment will be likely to be different in a therapeutic setting (155). More experiments especially clinical trials are needed to determine these parameters. Currently, on account of limited available information of helminth therapy, many problems are not clear such as whether other immune cells or cell subsets are involved in immunomodulation, as well as how helminth-induced type 2 immunity affects metabolic organs other than adipose tissue, including liver, skeletal muscle, gut and pancreas (162). Thus, there is a long way to clarify the mechanism and find more effective strategies against obesity and its associated diseases.

In addition, the emerging immunometabolism, provides a novel insight on addressing these scientific puzzles mentioned above. Accumulating evidence has indicated that helminth or its derived molecules can reprogram the metabolic events in macrophages of host, thereby modulating the anti-infective immunity and providing a moderate living environment for the growth and development of helminth (163). Nevertheless, only sporadic mechanistic investigations in the view of immunometabolism have been reported (164, 165). Several metabolites may be implicated in the inner workings of helminth infections and immunity. For example, SUCNR1 has recently been reported to increase the expression of the anti-inflammatory markers related to M2 macrophages in WD induced obesity (70). The enzyme aconitate decarboxylase 1 (ACOD1, originally named by immune responsive gene 1, IRG-1), is responsible for itaconate production through the decarboxylation of cis-aconitate in the TCA cycle. The latest studies have uncovered that ACOD1/itaconate axis links metabolism to immunity in macrophages (166), and has gained lots of interests in immunometabolism field and inflammatory disease models (167, 168). Of note, our lncRNA microarray analysis showed that larval *E. granulosus* infection can upregulate the expression of SUCNR1 and ACOD1 in adipose tissues of mice (26), which is paralleled with the M2 macrophage polarization post infection. Interestingly, a recent study reported that injection of soluble egg derived from *S. japonicum* exhibited inhibitory effects on the expression of lipogenesis-related genes in mice fed with WD, thereby contributing to the treatment for obesity-related fatty liver disease (169). These studies emphasize the intimate crosstalk between immune and metabolism of host and helminth or derived molecules. It is believed that immunometabolism can improve our understanding of the inherent mechanisms of host-helminth/derived molecules interplay, promising to contribute to a stream of innovative therapeutic avenues for obesity and its related Mets.

## Author Contributions

WP conceived the manuscript. MD, XY, YY, and WP wrote the manuscript. All authors have seen and approved the submitted version of this manuscript.

## Funding

Project support was provided in part by the National Natural Science Foundation of China (Nos. 81871670, 81800718, 82071184), Natural Science Foundation of Jiangsu Province (No. BK20201459), the Training Programs of Innovation and Entrepreneurship for College Students in Jiangsu Province (Nos. 202010313035Z, 202010313009), Jiangsu Planned Projects for Postdoctoral Research Funds (No. 2019K063) and Jiangsu Qinglan Project. The funders had no role in study design, data collection and analysis, decision to publish, or preparation of the manuscript.

## Conflict of Interest

The authors declare that the research was conducted in the absence of any commercial or financial relationships that could be construed as a potential conflict of interest.

## Publisher’s Note

All claims expressed in this article are solely those of the authors and do not necessarily represent those of their affiliated organizations, or those of the publisher, the editors and the reviewers. Any product that may be evaluated in this article, or claim that may be made by its manufacturer, is not guaranteed or endorsed by the publisher.
